# Associations between Language at 2 Years and Literacy Skills at 7 Years in Preterm Children Born at Very Early Gestational Age and/or with Very Low Birth Weight

**DOI:** 10.3390/children8060510

**Published:** 2021-06-16

**Authors:** Eveliina Joensuu, Petriina Munck, Sirkku Setänen, Jari Lipsanen, Mira Huhtala, Helena Lapinleimu, Suvi K. J. Stolt

**Affiliations:** 1 Department of Psychology and Logopedics, Faculty of Medicine, University of Helsinki, 00014 Helsinki, Finland; petriina.munck@gmail.com (P.M.); jari.lipsanen@helsinki.fi (J.L.); suvi.stolt@helsinki.fi (S.K.J.S.); 2 Department of Pediatrics and Adolescent Medicine, University of Turku and Turku University Hospital, 20014 Turku, Finland; sirkku.setanen@utu.fi (S.S.); lehela@utu.fi (H.L.); 3 Oncology and Radiotherapy, Turku University Hospital, 20014 Turku, Finland; mivall@utu.fi

**Keywords:** early language development, literacy skills, very preterm, very low birth weight, prematurely born children, longitudinal follow-up, regional cohort study, assessment

## Abstract

Preterm children (born <37 gestational weeks) who are born at very early gestational age (<32 weeks, very preterm, VP) and/or with very low birth weight (≤1500 g, VLBW) are at increased risk for language and literacy deficits. The continuum between very early language development and literacy skills among these children is not clear. Our objective was to investigate the associations between language development at 2 years (corrected age) and literacy skills at 7 years in VP/VLBW children. Participants were 136 VP/VLBW children and 137 term controls (a 6-year regional population cohort, children living in Finnish-speaking families). At 2 years of corrected age, language (lexical development, utterance length) was assessed using the Finnish version of the MacArthur–Bates Communicative Development Inventory and the Expressive Language Scale from Bayley scales of Infant Development, second edition. At 7 years, children’s literacy skills (pre-reading skills, reading, and writing) were evaluated. Statistically significant correlations were found in both groups between language development at 2 years and literacy skills at 7 years (*r*-values varied between 0.29 and 0.43, *p* < 0.01). In the VP/VLBW group, 33% to 74% of the children with early weak language development had weak literacy skills at 7 years relative to those with more advanced early language skills (11% to 44%, *p* < 0.001 to 0.047). Language development at 2 years explained 14% to 28% of the variance in literacy skills 5 years later. Language development at 2 years had fair predictive value for literacy skills at 7 years in the VP/VLBW group (area under the receiver operating characteristic (ROC) curve (AUC) values varied between 0.70 and 0.77, *p* < 0.001). Findings provide support for the continuum between very early language development and later language ability, in the domain of literacy skills in preterm children.

## 1. Introduction

Prematurely born (<37 gestational weeks) children born at very early gestational age (<32 gestational weeks, very preterm, VP) and/or with very low birth weight (≤1500 g, VLBW) are at increased risk for developmental impairments and learning deficits such as difficulties in early language development [1,2,3] and literacy skills [4,5,6,7], including pre-reading skills, reading, and writing. The gap to full-term controls in language and literacy skills is evident even in the absence of neurodevelopmental impairment (NDI), including cerebral palsy, hearing impairment, blindness, or severe cognitive impairment (intelligence quotient, IQ < 70) [8,9]. The goal of clinical follow-up is to identify weak development as early as possible to provide targeted intervention to improve developmental outcomes. Findings of recent longitudinal studies, although sparse, suggest that difficulties in language functions persist from early years through late childhood, up to the age of 13 years [3,10,11]. Previous investigations, including a recent study of a large French cohort [12], highlight the usefulness of a validated parent-reported measure, such as the MacArthur–Bates Communicative Development Inventories (CDI) [13], in assessing early language skills of children born VP/VLBW to predict developmental difficulties in language [2,3].

Although earlier studies have provided essential information regarding the continuum between early language skills and later language performance in children born VP/VLBW, far fewer reports have used literacy skills as an outcome measure [14,15,16,17]. Furthermore, most of the existing studies examining associations between language and literacy skills have been based on samples of school-aged children, not assessing very early childhood. To date, the earliest age point in a longitudinal setting has been reported by Pritchard et al. [17] who investigated the relations between school readiness domains, including language, at the corrected age (i.e., adjusted age, representing the age of the child from the expected date of delivery) of 4 years and later educational achievement at school age. To the best of our knowledge, the possible longitudinal associations between very early language development at 2 years and literacy skills at 7 years is an open question in this high-risk population.

In early clinical follow-up of prematurely born children, their development is often followed up to the age of 2 years. However, the language development of VP/VLBW children is not always assessed specifically. Clinicians evaluating early language development of children born VP/VLBW would benefit from the knowledge of whether lexical development and utterance length at 2 years of corrected age have predictive value for literacy outcome at 7 years in this vulnerable population, and whether there is a cost-effective way of identifying toddlers at potential risk for literacy deficits. To maximize the effects of early intervention, it is crucial to identify children with weak skills as early as possible.

In the current study, we analyze longitudinal associations between language skills at 2 years of corrected age and literacy skills at 7 years in a Finnish sample of children born VP/VLBW. In Finland, children begin formal schooling in the year in which they reach the age of 7 years. Finnish is a transparent language with a highly regular grapheme-phoneme correspondence, and thus, basic decoding skills are often acquired during the first year of school see e.g., [18]. In addition, more than one-third of Finnish first-graders can already read before entering school. Previous findings from longitudinal studies investigating Finnish children with a hereditary risk for dyslexia and their controls [19,20] suggest that features of early language development, including lexical development and utterance length, have predictive value for later reading acquisition [20,21]. In preterm children, this association has not been analyzed previously.

This study had three aims: (1) to evaluate the associations between language skills at 2 years of corrected age and literacy skills at the beginning of schooling, at 7 years, in a regional cohort of Finnish-speaking children born VP/VLBW and in their full-term controls; (2) to analyze how much early language skills explain the variance of literacy skills at 7 years; and (3) to assess the predictive value of language skills at 2 years for literacy skills at 7 years measured using area under the receiver operating characteristic (ROC) curve (AUC) values in VP/VLBW children and their controls.

## 2. Materials and Methods

### 2.1. Participants

This study is part of the multidisciplinary 6-year regional cohort study of prematurely born children called PIPARI (Development and Functioning of Very Low Birth Weight Infants from Infancy to School Age) [22,23]. The participants were children born <32 weeks of gestational age and/or with birth weight ≤1500 g in Turku University Hospital, Finland, in 2001–2006. From 2001 to 2003, the inclusion criteria were birth weight ≤1500 g and prematurity (<37 gestational weeks). From the beginning of 2004, the inclusion criteria were expanded to include all infants born <32 weeks of gestation, regardless of birth weight. At least one of the parents had to speak Finnish or Swedish, the two official languages in Finland. Children with severe congenital anomalies or diagnosed syndromes affecting their development were excluded.

**Figure 1:**
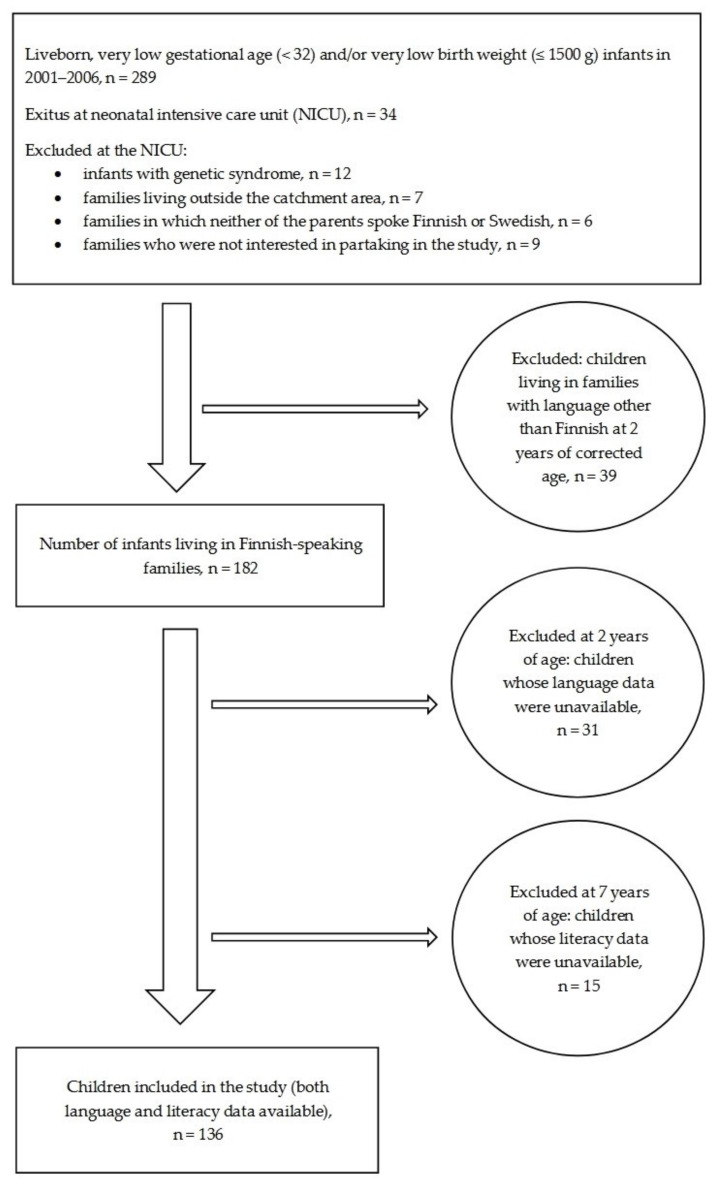
Flow chart of the prematurely born children born at very early gestational age (<32 weeks, very preterm, VP) and/or with very low birth weight (≤1500 g, VLBW) included in the study.

The present study sample consisted of 136 children born VP/VLBW living in Finnish-speaking families. The flow chart of the children born VP/VLBW participating in this study is presented in Figure 1. Neurodevelopmental impairment was determined if one or more of the following factors were present by the corrected age of 2 years: cerebral palsy, hearing impairment (threshold >40 dB), blindness, or severe cognitive impairment (Mental Developmental Index, MDI of the Bayley Scales of Infant Development II [24], BSID-II, <70 standard scores). In the PIPARI study, the age of VP/VLBW children was corrected for prematurity until the age of 2 years.

The control group consisted of healthy full-term (≤37 weeks of gestation) infants born in the same hospital between 2001 and 2004. They were recruited by asking the parents of the first boy and the first girl born in each week to take part in the study. If the family was not interested in partaking in the study, the parents of the next boy/girl were invited. The full-term controls were born ≥37 weeks of gestation, were not admitted to a neonatal care unit during the first week of life, and had at least one parent speaking either Finnish or Swedish. The exclusion criteria were any major congenital anomalies or chromosomal or genetic syndromes, the mother’s known use of illicit drugs or alcohol during pregnancy, and the infant’s birth weight being small for gestational age according to age- and gender-specific Finnish growth charts. In the present study, only those 136 children born VP/VLBW and those 137 controls who had data available from both the language assessment at 2 years of corrected age and literacy skills assessment at 7 years were included.

The PIPARI study protocol was approved by the Ethics Review Committee of the Hospital District of Southwest Finland in December 2000 and January 2012. After receiving oral and written information, all parents who agreed to participate provided written informed consent.

### 2.2. Assessment at 2 Years of Corrected Age

Language skills were assessed with the Finnish long-form version of the MacArthur–Bates Communicative Development Inventory (FinCDI, toddler version) [25], and the Expressive Language Score (ELS) from BSID-II. The FinCDI is a validated, parent-report measure evaluating the development of lexicon and grammar, including inflectional morphological skills. Variables of lexicon size and mean length of the three longest utterances (M3L) were used. Lexicon size is the number of words the parents estimated that their child uses, based on word lists (595 words). M3L is calculated in morphemes (i.e., the smallest units of language creating a difference in meaning) based on the three longest recent utterances the child has made. The ELS consists of 10 pictures and 5 objects that the child was asked to name in the testing situation.

### 2.3. Assessment at 7 Years

Reading precursors, reading, and writing ability were evaluated during the first weeks of grade 1 of primary school (a 6-week period from August to September during the school entrance year). Reading precursors assessed were phonological awareness and letter knowledge. To evaluate phonological awareness, three- to seven-letter words were presented phoneme by phoneme [26]. Children were instructed to mark one picture out of four alternatives that they thought would best match the word (max 9). To evaluate letter knowledge, the child was asked to name 29 uppercase letters presented in random order (max 29) [27]. In this study, the sum score of the tasks of phonological awareness and letter knowledge was used as the measure of precursors of reading (max 38).

Reading skills were evaluated using a short version of the Finnish reading test ARMI—a tool for assessing reading and writing skills in Grade 1 [27], consisting of a wordlist of two-syllable (seven words), three-syllable (two words), and five-syllable (one word) words. The child was asked to read the words aloud. The score for reading skills was the number of correctly read words (max 10). To evaluate writing skills, the child was asked to write 5 words and 8 pseudowords said aloud one word at a time [19]. The writing skills score was the total number of correctly written items (max 13).

### 2.4. Statistical Analyses

All analyses were run separately for all children born VP/VLBW, for preterm children without neurodevelopmental impairment, and for controls. Pearson’s correlation coefficient values were used to investigate the correlations between the continuous language and literacy variables measured at 2 and 7 years. All language and literacy variables were also categorized. The 10th percentile cut-off value was used to evaluate the association between early weak language skills at 2 years of corrected age and weak literacy skills at 7 years of age. For the FinCDI, the cut-off value was based on the normative sample, and for the other measures, the 10th percentile cut-off values were derived from the control group. Comparisons between categorical variables were done using cross-tabulation with the Chi-square test or Fisher’s exact test. Multiple variable linear regression analysis was conducted to assess how much 2-year language variables explain the variance in literacy skills at 7 years when the effect of background factors were taken into consideration. The dependent variables were reading precursors (sumscore of letter knowledge and phoneme synthesis), reading, and writing skills at 7 years. The independent variables were lexicon size, M3L, and ELS measured at 2 years. Since the independent variables were strongly correlated with each other, they were analyzed separately. Nine regression models were run: in the first three models, lexicon size was used as an independent variable; in the next three models, M3L; and in the last three models, ELS. In the preliminary analyses, the following background factors were associated with the outcome variables and were therefore included in the regression models: gestational age, mother’s self-reported reading difficulties, father’s self-reported reading difficulties, and paternal education. Maternal education was not included because in the preliminary analysis paternal education level correlated more strongly with the outcome variables. Due to multicollinearity between maternal and paternal education, only paternal education was included in the regression analyses. Lastly, the predictive value of early language development at 2 years for literacy skills at 7 years was analyzed using the AUC values. The AUC is the measure of the ability of a test to distinguish between classes [28]. The greater the AUC values, the better the prediction model. An area of 1. represents a perfect classifier, whereas a ROC curve no better than chance would have an area under the curve of 0.5. AUC values are interpreted as follows: excellent predictive value 0.90–1, good 0.80–0.90, fair 0.70–0.80, poor 0.60–0.70, and fail <0.60 [28]. All statistical analyses were performed using IBM^®^ SPSS^®^ Statistics for Windows, version 26.0. (IBM Corp., Armonk, NY, USA). Two-tailed *p*-values < 0.05 were considered statistically significant.

## 3. Results

### 3.1. Data Description

The background characteristics of the children are presented in Table 1. No statistically significant difference in background factors was found between the children born VP/VLBW who participated in the study and the VP/VLBW children living in Finnish-speaking families whose language and literacy data were unavailable (*n* = 46, 25%), except that there were more multiple births among participating children (36% of the study children vs. 16% of the dropouts, *p* = *0*.02).

Descriptive statistics for language and literacy variables were measured and group comparisons are presented in Table 2. A statistically significant difference between the groups was found in every language (*p*-values from 0.04 to < 0.001) and literacy variable (*p*-values from 0.002 to 0.003). When children with neurodevelopmental impairment were excluded, the group differences remained statistically significant in ELS and in every literacy variable.

### 3.2. Associations between Language Development at 2 years of Corrected Age and Literacy Skills at 7 Years

Statistically significant positive correlations were found in both groups between all variables measured at 2 and 7 years (*r*-values between 0.29 and 0.43, *p* < 0.01) (Table 3). When children with neurodevelopmental impairment were excluded, the correlations remained statistically significant. The *r*-values were slightly smaller for precursors of reading but remained the same or even slightly increased in reading and writing.

Based on the cross-tabulation, 33% to 74% of VP/VLBW children who had weak early language development (10th percentile) had also weak literacy skills at 7 years (see Table 4). The corresponding proportions for VP/VLBW children with typical language development at 2 years were 11% to 44%. In the controls, the corresponding proportions for children with weak language at 2 years were 15% to 83%. However, the results of the cross-tabulation were statistically significant only between weak lexicon size and weak reading skills and between weak M3L and weak reading and writing skills.

The regression models of the VP/VLBW group, including early lexicon size as a predictor, explained 23% of the variance in reading precursors, 27% of the variance in reading skills, and 17% of the variance in writing skills at 7 years (Table 5). In these models, early lexicon size and paternal education were statistically significant independent predictors. The regression models of the controls are presented in Appendix A.

The models including M3L as a predictor explained 27% of the variance in reading precursors, 28% of the variance in reading skills, and 16% of the variance in writing skills at 7 years (Table 6). M3L and paternal education were statistically significant independent predictors.

The models including ELS as a predictor (Table 7) explained 20% of the variance in reading precursors, 25% of the variance in reading skills, and 14% of the variance in writing skills at 7 years. ELS, paternal education, and mother’s self-reported reading difficulties were statistically significant independent predictors.

The exclusion of children with neurodevelopmental impairment did not alter the results. In the control group (Appendix A), the same models explained a smaller proportion of the variance in outcome relative to children born VP/VLBW.

In VP/VLBW group, the AUC values of language variables at 2 years regarding literacy skills at 7 years varied between 0.70 and 0.77 (*p* < 0.001) (Table 8). Exclusion of children with neurodevelopmental impairment did not significantly alter the results. In controls, the values varied between 0.62 and 0.73 (*p*-values from 0.18 to < 0.001), respectively.

## 4. Discussion

To the best of our knowledge, this is the first controlled follow-up study providing longitudinal information on the associations between very early language development at 2 years of corrected age and later literacy skills in VP/VLBW children and their controls. Significant correlations between every language and literacy variable were found both in the VP/VLBW group and in the control group. Most of the children born VP/VLBW with weak language skills at 2 years had also weak literacy skills at 7 years. Lexicon size, M3L, and ELS measured at 2 years were statistically significant predictors in the regression models explaining the variance in literacy skills, especially in the VP/VLBW group. Every language variable at 2 years had a fair predictive value for literacy skills 5 years later in children born VP/VLBW when measured using AUC values.

Previously, the associations between language and literacy ability in children born preterm have been analyzed at 4 years of age at the earliest [17]. In a longitudinal study consisting of 110 children born VP and 113 term controls, Pritchard and colleagues [17] found an association between school readiness domains including language at age 4 years, and literacy and numeracy skills at ages 6 and 9 years. In addition, in the study of Perez-Pereira et al. [30], morphosyntactic production and comprehension of syntactic structures at 5 years were associated with reading outcome at 9 years in preterm children. The knowledge of letters and words at 5 years [31] and phonological awareness and expressive and receptive language at 6 years [16] have been found to be associated with reading and writing outcome at 7 years [31] and at 8 years [16] in VP populations. In two previous cross-sectional studies [14,15], reading performance at 8 years in children born VP was correlated with lexical production and grammar comprehension [14] and with phonological awareness and rapid naming [15]. To date, it has been unclear whether an association between very early language development and later literacy outcome can be detected in VP/VLBW children. Our findings fill in this gap, suggesting that the association between language and literacy at 7 years of age is evident already at age 2 years in children born VP/VLBW.

In the present cohort, most children born VP/VLBW with small lexicon size, short M3L, and weak ELS at 2 years had weak literacy skills at 7 years compared with those with more advanced early language. In previous studies considering the continuum of language in children born VP/VLBW small lexicon size and short utterance length at 2 years have been shown to predict later language development [3,4,10,12]. Our study extends this knowledge to literacy skills. These results together emphasize the need for early screening of weak language development in the vulnerable group of VP/VLBW children

Another novel finding was that language skills at 2 years explained a significant amount of the variance in literacy skills 5 years later, especially in the VP/VLBW group. Thus, the present findings provide evidence for the existence of a continuum between language development at 2 years and literacy ability at 7 years in these children. This study offers an interesting perspective on the association of early language with later literacy skills in preterm children using the Finnish language, which has a highly regular grapheme–phoneme correspondence. Different language versions of the CDI have been shown to be a cost-effective way of identifying small lexicon and short utterance length at 2 years [2,3,4,12]. In this study, the variables of the FinCDI were even stronger predictors for later literacy skills than ELS, which is a performance-based subtest of BSID-II [24]. Thus, our results support the view that parents can provide valuable information on early language development of their children, when structured, validated measure, such as CDI, is used.

Paternal education was a significant background variable in the regression models, especially in children born VP/VLBW. For the controls, the effect of paternal education was not as clear. The effects of paternal education are less studied than those of maternal education. However, in previous studies regarding the same PIPARI cohort [22,23], paternal education was found to relate also to precursors of reading at 5 years [5], and to verbal comprehension at 11 years [32] in children born VP/VLBW. Our findings suggest that fathers may have a significant role in supporting the language development of preterm children in the home environment during childhood years, at least in societies which emphasize the role of both parents in early childhood care, as in Finland.

In this study, lexicon size, M3L, and ELS measured at 2 years had fair predictive value (AUC values varied between 0.70 and 0.77, *p* < 0.001) for literacy skills 5 years later in the VP/VLBW group. The explaining value of early language at 2 years of age for literacy performance at school age has been investigated previously in full-term populations, e.g., in children with a familial risk of dyslexia [19,20]. Parallel results have been noted for late talkers, i.e., children with small expressive lexicon at 2 years but with an absence of cognitive delay or any other neurological condition explaining the slow language acquisition [33,34]. Late talkers perform consistently lower on language and literacy tasks at school age and even in adolescence than their peers [33,34,35]. In the present study, the predictive value of early language at 2 years of age for literacy skills at 7 years was established for the first time in the vulnerable population of preterm children born at very early gestational age (<32) and/or with very low birth weight (≤1500 g). Comparable findings detected in different populations support the view that very small lexicon size and/or very short utterance length at 2 years of age are risk factors for later language and literacy deficits after controlling for background factors. Furthermore, the predictive value of early language skills for later literacy outcome was better in children born VP/VLBW than in controls. This finding may be explained by the fact that the VP/VLBW sample included more children with early weak language skills relative to controls [3]. These results may also reflect the persistence of language-related difficulties among children born VP/VLBW with early weak language.

This study has several implications. First, it shows very clear longitudinal associations between very early language skills and later literacy outcome in preterm children. Thus, our findings propose the clinical importance of screening language skills of preterm children born at very early gestational age and/or with very low birth weight at 2 years of corrected age. Our findings highlight the usefulness of the validated parent-reported form, such as CDI [13,25] in the follow-up of the vulnerable group of high-risk prematurely born VP/VLBW children for identifying children at risk for later literacy deficits. Identifying developmental problems as early as possible is important, since it enables targeted early interventions and support. In addition, standardized parental report forms, such as CDI, may promote parents’ active involvement in observing and supporting the language development of their preterm-born child. Our results provide information also for the educational professionals working with school-aged children born VP/VLBW showing the higher percentage of weak pre-reading, reading and writing skills in this population when compared with full-term control children.

Strengths of the study include its longitudinal design with a well-defined cohort of children born VP/VLBW together with a control group born in the same hospital. The longitudinal data from altogether 274 children provided a great possibility to assess the associations between early language development and later literacy performance. Both a validated parent-report form [13,25] and a test-based measure [24] were used to gather information on early lexical and grammatical development. The use of different types of method to assess early language development strengthened our findings. In our study, the phonological awareness task, which included three to seven-letter words said aloud phoneme by phoneme, also relates to working memory and actually measures both domains. However, the participants also had visual aid: at the same time as they heard the phonemes, they saw pictures of the correct word and three other alternatives. The participants had to mark one picture out of four alternatives they thought would best match the word. This might have reduced the burden of the working memory during the task. As a limitation, measures used in the study provided information on expressive language only. Information on receptive language would have provided an even more comprehensive view of early language development. This should be taken into consideration when applying these results to a clinical context.

## 5. Conclusions

Language development is essential for academic learning of children starting school. It is important to recognize potential risks for learning disorders as early as possible. Our study shows, for the first time, that problems in literacy skills at the beginning of formal schooling at 7 years of age may be identified already at age 2 years in preterm children born at very early gestational age and/or with very low birth weight. Early identification enables early interventions for those preterm children at risk for later literacy deficits. If concern arises regarding the early language ability of preterm children based on the results of a parental report form, such as CDI, a broader assessment of language skills by a speech-language pathologist is recommended. We emphasize the need for further studies (randomized controlled trials) regarding effective early interventions for VP/VLBW children at risk for literacy deficits.

## Figures and Tables

**Table 1:** Background characteristics of very preterm/very low birth weight (VP/VLBW) children and full-term controls. Numbers (percentages) are shown. If mean (standard deviation) [minimum, maximum] are presented, they are indicated separately.

Characteristic	Children Born VP/VLBW	Controls
	(*n* = 136)	(*n* = 137)
	*n* (%)	*n* (%)
Gestational age (weeks); M (SD), (min., max)	28.9 (2.7) (23.0, 35.9)	40.2 (1.2) (37.1, 42.3)
Birth weight (grams); M (SD) (min., max)	1116 (303) (400, 1820)	3663 (442) (2830, 4980)
Small for gestational age ^a,^	39 (29)	0
Prenatal corticosteroids	129 (95)	–
Multiple birth	49 (36)	0
Male	83 (61)	67 (49)
Bronchopulmonary dysplasia	22 (8)	–
Laser-treated retinopathy of prematurity	4/127 ^d^ (3),	–
Neurodevelopmental impairment	13 (10)	0
Mental Developmental Index <70	3/134 ^d^ (2)	0
Cerebral palsy	9 (7)	0
Hearing impairment (threshold >40)	4 (3)	0
Visual impairment	0	0
Brain pathology, MRI at term age ^b^		–
Normal finding or minor abnormality	94/135 ^d^ (69)	–
Major abnormality	41/135 ^d^ (30)	–
Maternal education ^c^		
High	64/127 ^d^ (47)	43 (31)
Intermediate	52/127 ^d^ (38)	70 (51)
Low	11/127 ^d^ (8)	24 (18)
Paternal education ^c^		
High	36/126 ^d^ (27)	36 (26)
Intermediatel	80 (59)	72 (53)
Low	10 (7)	29 (21)

^a^ Small for gestational age was defined as a birth weight < −2.0 SD, according to the age- and gender-specific Finnish growth charts. ^b^ See specific magnetic resonance imaging (MRI) protocol and details about the classification elsewhere [29]. ^c^ High is defined as a Bachelor’s degree, Master’s degree, or Doctoral degree; Intermediate is defined as high school or vocational school; Low is defined as primary or lower secondary or less. ^d^ The percentages were calculated from the data available. VP/VLBW = very preterm (<32 gestational weeks)/very low birth weight (≤1500 g).

**Table 2:** Descriptive statistics and group comparisons for the language variables at 2 years of age and literacy variables at 7 years of age for all VP/VLBW children, for VP/VLBW children without neurodevelopmental impairment (NDI), and for full-term controls.

	VP/VLBW Children	Controls	Group Comparison for the Mean		VP/VLBW Children	Controls
Measure	Mean (SD) (min, max)	Mean (SD) (min, max)	95% CI	*p*	Weak skills *n* (%)	Weak skills *n* (%)
Language at 2 years						
Lexicon size	236 (159) (4, 574)	281 (164) (9, 581)	5.87 to 82.76	0.017	21 (15%)	6 (4%)
M3L	5 (3) (1, 14)	6 (4) (1, 21)	0.02 to 1.61	0.036	26 (21%)	18 (14%)
ELS	9 (5) (0, 15)	11 (5) (0, 15)	0.86 to 3.31	<0.001	21 (17%)	13 (10%)
Literacy skills at 7 years						
Reading precursors	30 (8) (3, 38)	33 (6) (11, 38)	1.08 to 4.56	0.002	27 (20%)	13 (10%)
Reading	4 (4) (0, 10)	6 (4) (0, 10)	0.55 to 2.57	0.003	49 (36%)	25 (18%)
Writing	3 (4) (0, 13)	4 (4) (0, 13)	0.70 to 2.65	0.001	64 (48%)	45 (33%)
VP/VLBW children without NDI						
Language at 2 years						
Lexicon size	247 (155) (4, 574)		−5.08 to 73.1	0.087	16 (13%)	
M3L	5 (3) (1, 14)		−0.20 to 1.43	0.138	20 (18%)	
ELS	9 (5) (0, 15)		0.57 to 3.05	0.004	17 (15%)	
Literacy skills at 7 years						
Reading precursors	31 (8) (4, 38)		0.64 to 4.06	0.007	23 (19%)	
Reading	4 (4) (0, 10)		0.42 to 2.50	0.006	36 (44%)	
Writing	3 (4) (0, 13)		0.59 to 2.60	0.002	60 (49%)	

VP/VLBW = very preterm (<32 gestational weeks)/very low birth weight (≤1500 g); NDI = Neurodevelopmental Impairment; *(SD)* = Standard Deviation; (min, max) = minimum and maximum; *n* = number; (%) = percentages; 95% CI = Confidence Interval for the mean; *p* = significance level; M3L = mean length of the three longest utterances value; ELS = Expressive Language Score.

**Table 3:** Pearson correlation coefficient values (*r*-values) between language measures at 2 years and literacy measures at 7 years of age for all VP/VLBW children, for VP/VLBW children without NDI, and for the full term controls.

	7 y		
	Reading Precursors	Reading Skills	Writing Skills
2 y			
Children born VP/VLBW			
Lexicon size	0.37 **	0.40 **	0.33 **
M3L	0.43 **	0.41 **	0.31 **
ELS	0.36 **	0.39 **	0.29 **
Children born VP/VLBW without NDI			
Lexicon size	0.33 **	0.42 **	0.34 **
M3L	0.39 **	0.43 **	0.32 **
ELS	0.30 **	0.39 **	0.31 **
Controls			
Lexicon size	0.32 **	0.39 **	0.32 **
M3L	0.34 **	0.39 **	0.36 **
ELS	0.29 **	0.38 **	0.33 **

** Correlation is significant at the 0.01 level (2-tailed). VP/VLBW = very preterm (<32 gestational weeks)/very low birth weight (≤1500 g); NDI = neurodevelopmental impairment; y = years.

**Table 4:** Results of the cross-tabulation with Chi-square or Fisher’s exact tests of the associations between weak lexicon size (10th percentile, <30 words), weak M3L (10th percentile, <2.06), weak ELS (10th percentile, <1.60) measured at 2 years, and weak reading precursors (10th percentile, <25), weak reading (<10th percentile, 0 words), and weak writing (10th percentile, 0 words) measured at 7 years. Results for all VP/VLBW children, for VP/VLBW children without NDI, and for full-term controls are presented.

	Weak Reading Precursors at 7 years					
	Children born VP/VLBW		Children without NDI		Controls	
Measured at 2 years	n (%)	*p*	n (%)	*p*	n (%)	*p*
Lexicon size		0.001		0.039		0.101
Weak	10 (48%)		6 (38%)		2 (33%)	
Normal	17 (15%)		17 (16%)		11 (8%)	
M3L		<0.001		<0.001		0.058
Weak	13 (50%)		9 (45%)		4 (22%)	
Normal	11 (11%)		11 (12%)		8 (7%)	
ELS		0.037		0.169		0.325
Weak	7 (33%)		5 (29%)		2 (15%)	
Normal	16 (15%)		15 (15%)		10 (8%)	
	Weak reading skills at 7 years					
Lexicon size		0.007		0.068		0.301
Weak	13 (62%)		9 (56%)		2 (33%)	
Normal	36 (31%)		35 (33%)		23 (18%)	
M3L		0.009		0.058		0.013
Weak	14 (54%)		10 (50%)		7 (39%)	
Normal	27 (27%)		26 (28%)		17 (15%)	
ELS		0.08		0.101		0.253
Weak	11 (51%)		9 (53%)		4 (31%)	
Normal	34 (32%)		32 (32%)		20 (16%)	
Lexicon size		0.019		0.027		0.019
Weak	14 (74%)		12 (75%)		5 (83%)	
Normal	50 (44%)		48 (45%)		40 (31%)	
M3L		0.005		0.004		<0.001
Weak	17 (71%)		15 (75%)		14 (78%)	
Normal	39 (39%)		37 (40%)		28 (24%)	
ELS		0.028		0.019		0.268
Weak	14 (70%)		13 (77%)		6 (46%)	
Normal	46 (44%)		44 (44%)		38 (31%)	

M3L = mean length of the three longest utterances value; ELS = Expressive Language Score; VP/VLBW = very preterm (<32 gestational weeks)/very low birth weight (≤1500 g); NDI = neurodevelopmental impairment.

**Table 5:** Results of multiple variable linear regression analysis with reading precursors, reading and writing skills at 7 years of age as dependent variables, and with lexicon size at 2 years of corrected age and background factors as independent variables. Results of VP/VLBW children are presented.

	Reading Precursors			Reading			Writing		
	*b*	95% CI	*p*	*b*	95% CI	*p*	*b*	95% CI	*p*
Gestational age	−0.05	−0.66 to 0.38	0.589	0.04	−0.20 to 0.32	0.642	0.03	−0.20 to 0.27	0.764
Reading difficulties									
Mothers	0.15	−0.40 to 9.50	0.071	0.10	−0.93 to 4.01	0.223	0.07	−1.29 to 3.25	0.462
Fathers	−0.07	−5.63 to 2.39	0.432	−0.02	−2.28 to 1.73	0.791	−0.07	−2.73 to 0.95	0.412
Paternal education	0.25	1.47 to 7.56	0.004	0.31	1.42 to 4.46	<0.001	0.20	0.26 to 3.05	0.024
Lexicon size	0.31	0.01 to 0.03	0.001	0.32	0.004 to 0.01	<0.001	0.30	0.002 to 0.01	0.001
Fit statistics									
F	7.0			9.0			5.0		
P for F	<0.001			<0.001			0.001		
R^2^	0.23			0.27			0.17		
ΔR^2^	0.20			0.24			0.13		

VP/VLBW = very preterm (<32 gestational weeks)/very low birth weight (≤1500 g); F = value of F-statistic; *p* = significance value; R^2^ = coefficient of determination; ΔR^2^ = adjusted coefficient of determination.

**Table 6:** Results of multiple variable linear regression analysis with reading precursors, reading, and writing skills at 7 years of age as dependent variables, and with M3L at 2 years of corrected age and background factors as independent variables. Results of VP/VLBW children are presented.

	Reading Precursors			Reading			Writing		
	*b*	95% CI	*p*	*b*	95% CI	*p*	*b*	95% CI	*p*
Gestational age	−0.06	−0.72 to 0.34	0.484	0.03	−0.22 to 0.32	0.711	0.03	−0.21 to 0.29	0.762
Reading difficulties									
Mothers	0.12	−1.47 to 8.67	0.163	0.08	−1.35 to 3.82	0.351	0.07	−1.49 to 3.30	0.472
Fathers	−0.06	−5.51 to 2.63	0.491	−0.02	−2.28 to 1.87	0.842	−0.08	−2.78 to 1.07	0.294
Paternal education	0.25	1.50 to 7.66	0.004	0.32	1.44 to 4.57	<0.001	0.21	0.27 to 3.18	0.021
M3L	0.37	0.57 to 1.54	<0.001	0.35	0.25 to 0.75	<0.001	0.26	0.08 to 0.55	0.008
Fit statistics									
F	8.0			8.5			4.0		
P for F	<0.001			<0.001			0.003		
R^2^	0.27			0.28			0.16		
ΔR^2^	0.23			0.25			0.12		

M3L = mean length of the three longest utterances value; VP/VLBW = very preterm (<32 gestational weeks)/very low birth weight (≤1500 g); F = value of F-statistic; *p* = significance value; R^2^ = coefficient of determination; ΔR^2^ = adjusted coefficient of determination.

**Table 7:** Results of multiple variable linear regression analysis with reading precursors, reading, and writing skills at 7 years of age as dependent variables, and with ELS at 2 years of corrected age and background factors as independent variables. Results of VP/VLBW children are presented.

	Reading Precursors			Reading			Writing		
	*b*	95% CI	*p*	*b*	95% CI	*p*	*b*	95% CI	*p*
Gestational age	−0.01	−0.58 to 0.50	0.901	0.07	−0.15 to 0.39	0.401	0.06	−0.17 to 0.33	0.531
Reading difficulties									
Mothers	0.18	0.24 to 10.48	0.039	0.13	−0.58 to 4.56	0.132	0.11	−0.98 to 3.72	0.253
Fathers	−0.06	0.5.75 to 2.71	0.501	−0.02	−2.33 to 1.91	0.824	−0.08	−2.80 to 1.08	0.384
Paternal education	0.20	0.46 to 7.10	0.029	0.27	0.88 to 4.19	0.003	0.18	−0.09 to 2.93	0.069
ELS	0.26	0.13 to 0.72	0.005	0.28	0.08 to 0.38	0.002	0.21	0.01 to 0.28	0.029
Fit statistics									
F	6.0			7.0			3.4		
P for F	<0.001			<0.001			0.007		
R^2^	0.20			0.25			0.14		
ΔR^2^	0.17			0.21			0.10		

ELS = Expressive Language Score; VP/VLBW = very preterm (<32 gestational weeks)/very low birth weight (≤1500 g); F = value of F-statistic; *p* = significance value; R^2^ = coefficient of determination; ΔR^2^ = adjusted coefficient of determination.

**Table 8:** Area under the receiver operating characteristic (ROC) curve (AUC) values for weak reading precursors (sum score < 25, 10th percentile), weak reading (0 words, 10th percentile), and weak writing (0 words, 10th percentile) skills at 7 years with lexicon size/M3L/ELS at 2 years as predictor variables.

	AUC Value of Lexicon Size	95% CI	*p*
Children born VP/VLBW			
Reading precursors	0.70	0.58 to 0.83	0.001
Reading	0.72	0.63 to 0.81	<0.001
Writing	0.72	0.64 to 0.80	<0.001
Controls			
Reading precursors	0.65	0.50 to 0.80	0.081
Reading	0.67	0.56 to 0.78	0.009
Writing	0.69	0.60 to 0.78	<0.001
Children born VP/VLBW			
Reading precursors	0.77	0.66 to 0.89	<0.001
Reading	0.74	0.65 to 0.83	<0.001
Writing	0.73	0.64 to 0.82	<0.001
Controls			
Reading precursors	0.73	0.58 to 0.87	0.009
Reading	0.65	0.51 to 0.78	0.029
Writing	0.73	0.64 to 0.82	<0.001
Children born VP/VLBW			
Reading precursors	0.72	0.61 to 0.82	0.001
Reading	0.71	0.62 to 0.80	<0.001
Writing	0.74	0.65 to 0.83	<0.001
Controls			
Reading precursors	0.62	0.44 to 0.80	0.182
Reading	0.64	0.52 to 0.77	0.029
Writing	0.71	0.62 to 0.80	<0.001

AUC values are interpreted as follows: excellent predictive value 0.90–1, good 0.80–0.90, fair 0.70–0.80, poor 0.60–0.70, and fail < 0.60 [28]. AUC = Area Under the ROC Curve; M3L = mean length of the three longest utterances value; ELS = Expressive Language Score; VP/VLBW = very preterm (<32 gestational weeks)/very low birth weight (≤1500 g); NDI = neurodevelopmental impairment.

## Data Availability

This manuscript is based on health data. Access to these data is regulated by Finnish legislation and Findata, the Health and Social Data Permit Authority. The disclosure of data to third parties without explicit permission from Findata is prohibited. Only those fulfilling the requirements established by Finnish legislation and Findata for viewing confidential data are able to access the data. See https://www.findata.fi/en/about-us/data-protection-and-the-processing-of-personal-data/ (accessed on 29 May 2021).

## Appendix A

**Table A1:** Results of multiple variable linear regression analysis with reading precursors, reading, and writing skills at 7 years of age as dependent variables, and with lexicon size at 2 years of age and background factors as independent variables. Results of full-term controls are presented.

	Reading Precursors			Reading			Writing		
	*b*	95% CI	*p*	*b*	95% CI	*p*	*b*	95% CI	*p*
Gestational age	0.01	−0.87 to 0.94	0.94	0.02	−0.53 to 0.65	0.81	−0.04	−0.78 to 0.52	0.73
Reading difficulties									
Mothers	0.08	−2.67 to 3.90	0.38	0.06	−0.85 to 3.43	0.45	0.13	−0.58 to 4.12	0.22
Fathers	−0.10	−6.62 to 1.95	0.25	−0.02	−3.21 to 2.36	0.80	−0.04	−3.75 to 2.38	0.66
Paternal education	0.06	−1.36 to 3.45	0.47	0.19	0.38 to 3.51	0.029	0.12	−0.50 to 2.94	0.26
Lexicon size	0.38	0.01 to 0.19	<0.001	0.39	0.01 to 0.15	<0.001	0.34	0.005 to 0.01	<0.001
Fit statistics									
F	4.2			5.3			3.3		
P for F	0.001			<0.001			0.008		
R^2^	0.15			0.19			0.12		
ΔR^2^	0.12			0.15			0.09		

**Table A2:** Results of multiple variable linear regression analysis with reading precursors, reading, and writing skills at 7 years of age as dependent variables, and with M3L at 2 years of age and background factors as independent variables. Results of full-term controls are presented.

	Reading Precursors			Reading			Writing		
	*b*	95% CI	*p*	*b*	95% CI	*p*	*b*	95% CI	*p*
Gestational age	0.005	−0.90 to 0.94	0.95	0.02	−0.54 to 0.67	0.62	−0.04	−0.80 to 0.51	0.66
Reading difficulties									
Mothers	0.03	−2.80 to 3.80	0.77	0.09	−1.02 to 3.33	0.63	0.12	−0.66 to 4.03	0.16
Fathers	−0.10	−6.70 to 1.90	0.27	−0.03	−3.32 to 2.37	0.72	−0.04	−3.80 to 2.32	0.63
Paternal education	0.05	−1.80 to 3.04	0.61	0.17	0.02 to 3.22	0.04	0.09	−0.82 to 2.62	0.30
M3L	0.34	0.30 to 0.91	<0.001	0.38	0.26 to 0.67	<0.001	0.36	0.24 to 0.68	<0.001
Fit statistics									
F	3.4			5.6			4.1		
P for F	0.006			<0.001			0.002		
R^2^	0.13			0.19			0.16		
ΔR^2^	0.09			0.16			0.12		

M3L = mean length of the three longest utterances value.

**Table A3:** Results of multiple variable linear regression analysis with reading precursors, reading, and writing skills at 7 years of age as dependent variables, and with ELS at 2 years of age and background factors as independent variables. Results of full-term controls are presented.

	Reading Precursors			Reading			Writing		
	*b*	95% CI	*p*	*b*	95% CI	*p*	*b*	95% CI	*p*
Gestational age	0.01	−0.86 to 0.99	0.88	0.02	−0.52 to 0.68	0.80	−0.03	−0.79 to 0.53	0.70
Reading difficulties									
Mothers	0.04	−2.61 to 4.15	0.65	0.11	−0.72 to 3.65	0.38	0.14	−0.40 to 4.34	0.10
Fathers	−0.09	−6.64 to 2.13	0.31	−0.02	−3.19 to 2.48	0.81	−0.04	−0.370 to 2.50	0.68
Paternal education	0.06	−1.60 to 3.32	0.49	0.19	0.20 to 3.38	0.03	0.11	−0.70 to 2.80	0.22
ELS	0.29	0.14 to 0.59	0.002	0.38	0.19 to 0.48	<0.001	0.35	0.16 to 0.47	<0.001
Fit statistics									
F	2.5			5.5			3.7		
P for F	0.04			<0.001			0.004		
R^2^	0.09			0.20			0.14		
ΔR^2^	0.06			0.16			0.10		

ELS = Expressive Language Score.
